# Integrin Signaling in Cancer Cell Survival and Chemoresistance

**DOI:** 10.1155/2012/283181

**Published:** 2012-04-11

**Authors:** Fawzi Aoudjit, Kristiina Vuori

**Affiliations:** ^1^Pavillon CHUL, Centre de Recherche du CHUQ and Faculté de Médecine, Université Laval, Québec, QC, Canada G1V 4G2; ^2^Cancer Research Center, Sanford-Burnham Medical Research Institute, La Jolla, CA 92037, USA

## Abstract

Resistance to apoptosis and chemotherapy is a hallmark of cancer cells, and it is a critical factor in cancer recurrence and patient relapse. Extracellular matrix (ECM) *via* its receptors, the integrins, has emerged as a major pathway contributing to cancer cell survival and resistance to chemotherapy. Several studies over the last decade have demonstrated that ECM/integrin signaling provides a survival advantage to various cancer cell types against numerous chemotherapeutic drugs and against antibody therapy. In this paper, we will discuss the major findings on how ECM/integrin signaling protects tumor cells from drug-induced apoptosis. We will also discuss the potential role of ECM in malignant T-cell survival and in cancer stem cell resistance. Understanding how integrins and their signaling partners promote tumor cell survival and chemoresistance will likely lead to the development of new therapeutic strategies and agents for cancer treatment.

## 1. Introduction

Integrins are *α*/*β* heterodimeric membrane receptors that mediate cell-cell interactions and cell attachment to extracellular matrix (ECM). In addition to their role as cell adhesion molecules, ligation of integrins with ECM ligands induces a variety of intracellular signals and regulates several cellular responses including migration, differentiation, and proliferation [1–3]. Moreover, integrins also modulate programmed cell death or apoptosis. Most notably, many types of normal cells are absolutely dependent on proper ECM-integrin ligation for their survival. In cell culture experiments, lack of attachment of endothelial and epithelial cells to a proper matrix protein has been shown to induce a form of apoptosis that was termed anoikis [4, 5]. Since these ground-breaking studies, the role of integrin-ECM interactions in regulating cellular life and death has been the focus of extensive studies in the last decade. An intense area of research includes the understanding of integrin prosurvival function in the modulation of the chemotherapeutic response of cancer cells. Anticancer drugs used in chemotherapy are thought to exert their cytotoxic effects partially *via* induction of apoptosis [6]. Thus, a prevailing hypothesis is that cancer cell's resistance to apoptosis contributes to the development of drug resistance, which is an important factor in clinical relapse of cancer patients treated with chemotherapy. Initial studies have reported that integrin-ECM interactions can protect small cell lung cancer cells [7], multiple myeloma cell lines [8], and glioma cell lines [9] from drug-induced apoptosis. Further studies have extended the role of integrins in chemoresistance to other cancer cell types including various hematological malignancies and to several different classes of chemotherapeutic agents [10–14]. Integrins also provide survival advantage against death receptor-mediated apoptosis suggesting that they can promote cancer immune escape [15–19].

Herein, we will briefly review the role of integrins in apoptosis signaling and will discuss the major findings as to how the integrins may play a key role in the resistance of cancer cells to apoptosis and chemotherapy.

## 2. Programmed Cell Death (Apoptosis)

There are two major cellular death pathways that transduce the effects of various death inducers, including anticancer chemotherapeutic drugs. The extrinsic death pathway is mediated through cell death receptors of the TNF receptor family, such as the Fas receptor, whereas the intrinsic death pathway proceeds through the mitochondria [20–22]. Ligation of Fas with its ligand Fas-L induces Fas receptor aggregation, which in turn recruits the cytosolic adapter protein FADD to form the death inducing signaling complex (DISC). Caspase-8 is then recruited to the DISC and gets activated through aggregation and proteolytic cleavage. Activated caspase-8 in turn leads to the activation of executioner caspases such as caspase-3 [20, 22].

 The mitochondrial cell death is regulated by a balance between pro- and antiapoptotic Bcl-2 family of proteins [23–25]. Apoptotic stimuli that activate the mitochondrial death pathway lead to the activation of Bcl-2 proapoptotic proteins and inactivation of the Bcl-2 antiapoptotic proteins. Consequently, proapoptotic Bcl-2 proteins such as Bax/Bak become activated, which will cause the permeabilization of the mitochondria. This in turn leads to the release of apoptotic factors from mitochondria, among which is cytochrome c. Following its release, cytosolic cytochrome c binds to the adaptor protein Apaf-1, which in the presence of dATP recruits procaspase-9, thereby forming the apoptosome complex and leading to the activation of caspase-9. Activated caspase-9 then activates executioner caspases [20–25].

 In the so-called type II cells, activation of caspase-8 at the DISC is weak upon engagement of the extrinsic death pathway, and the apoptotic signal becomes amplified by the mitochondrial death pathway. In type II cells, activated caspase-8 at the DISC cleaves the proapoptotic Bcl-2 protein Bid, which then translocates to the mitochondria and activates Bax, leading to mitochondria permeabilization and subsequent activation of caspase-9. Caspase-9 then functions in concert with caspase-8, activating the executioner caspases [20–22].

 Initial studies have indicated that both of these apoptotic pathways can be important for the induction of apoptosis by chemotherapeutic agents. However, it is now clear that the mitochondrial death pathway is involved in the apoptotic action of most of the chemotherapeutic agents [6, 26, 27]. As it will be discussed below, integrins are able to modulate both the intrinsic and extrinsic apoptotic pathways.

## 3. Integrins and Cell Signaling

Integrins are cell surface membrane receptors composed of *α* and *β* chain heterodimers with short cytoplasmic tails devoid of any enzymatic activity. There are 18 different *α* chains and 8 *β* subunits in humans, which associate in pairs to give rise to at least 24 distinct *α*/*β* integrin heterodimers [28]. The *β*1 integrin subfamily is composed of 12 members as defined by the participating *α* subunit (*α*1–*α*12), is widely expressed, and constitutes a major class of integrins that mediate cell interactions with matrix proteins. The *α*1*β*1 and *α*2*β*1 integrins are major collagen receptors, whereas *α*4*β*1 and *α*5*β*1 integrins bind fibronectin, and *α*3*β*1 and *α*6*β*1 are receptors for laminins [28].

 Upon ligand binding, integrins form clusters on the cell surface at cellular sites termed focal adhesions that act not only as structural links between the ECM and the actin cytoskeleton but also as sites of signal transduction from the ECM to intracellular signaling pathways [1, 29, 30]. Focal adhesion kinase (FAK), integrin-linked kinase (ILK), and Src kinases have all been shown to be activated by integrin ligand binding [1, 29–31]. The ability of integrins to regulate apoptosis is likely due to their capacity to activate the cell survival signaling pathways further downstream of these cytoplasmic protein kinases, composed of phosphatidylinositol 3′-kinase (PI 3-kinase) and the serine/threonine kinase AKT, as well as the mitogen-activated protein kinase/extracellular regulated kinase (MAPK/ERK). The signaling events by which integrins activate these survival pathways are complex and may be tissue specific, but the dual kinase complex of FAK/Src has been shown to be involved in the activation of these survival pathways [1, 32].

## 4. Role of Integrin Signaling in Drug Resistance

Cultured cancer cells of epithelial origin typically are able to survive when denied attachment, suggesting that integrin ligand binding is no longer required to protect cancer cells from anoikis [33, 34]. Accordingly, resistance to anoikis contributes to anchorage-independent growth properties of cancer cells. Integrin-mediated cell attachment, however, has been shown to be required for cancer cell invasion and metastasis [2, 35, 36], and survival under various death-inducing conditions [34]. Most notably, the implication of integrin-ECM interactions in cell survival and resistance to chemotherapy seems to be a general phenomenon and occurs in multiple types of solid cancers including breast, lung, prostate, ovary, pancreatic and colon cancers, as well as in hematological malignancies, as reviewed below.

### 4.1. Solid Tumors

#### 4.1.1. Breast Cancer

We have shown in breast cancer cell lines MDA-MB-231 and MDA-MB-435 that ligation of *β*1 integrins inhibits apoptosis induced by paclitaxel and vincristine, two microtubule-directed chemotherapeutic agents widely used in the therapy of breast cancer [37]. We showed that attachment of MDA-MB-231 cells to fibronectin and to type I collagen *via* *α*5*β*1 and *α*2*β*1 integrins, respectively, significantly reduced drug-induced apoptosis. However, neither of these integrins/ligands had any effect on the survival of the MDA-MB-435 cells. In contrast, we found that it is the laminin-1-binding integrin *α*6*β*1 that mediates the protective effect against drug-induced apoptosis in these cells. It is noteworthy that more recent studies have found that the MDA-MB-435 originates from melanoma rather than from breast cancer [38] suggesting that *β*1 integrin can also protect melanoma cells from drug-induced apoptosis. Our results further indicated that the protective effect of *β*1 integrin in these cancer cells is mediated *via* activation of the PI 3-kinase/AKT pathway, which prevented the downregulation of Bcl-2 protein levels and inhibited drug-induced cytochrome c release upon drug treatment. These results indicate that *β*1 integrins inhibit drug-induced apoptosis at the level of the mitochondria. Importantly, laminin and fibronectin have also been shown to protect MDA-MB-231 cells and A549 lung cancer cells from radiation-induced apoptosis and from the cytotoxic drug ukrain [39].

 More recently, the Hemler laboratory reported that resistance of ErbB2-positive breast cancer cells to anti-Erb2 agents can be overcome by disrupting cell adhesion [40]. ErbB2 is an oncogene associated with higher grades of breast carcinomas. It is a member of the epidermal growth factor receptor family, also known as the human epidermal growth factor receptor 2 (HER2) [41]. Hemler and coworkers showed that adhesion of human Erb2-positive breast cancer cells to laminin-5 provided significant resistance to trastuzumab and lapatinib, an antibody and a small-molecule, respectively, that target ErbB2. The laminin-5 effect is mediated *via* *α*3*β*1 and *α*6*β*4 integrins and the associated tetraspanin CD151 receptor, and *via* downstream signaling through AKT, ERK1/2, and FAK. Another study reported that expression of *β*1 integrins is inversely correlated with the sensitivity of HER-2-positive breast cancer cells to trastuzumab treatment, suggesting that the *β*1 integrin is a potentially novel independent prognostic biomarker of trastuzumab response in HER-2-positive metastatic breast cancer patients [42]. ErbB-2 also mediates transcriptional upregulation of the *α*5*β*1 fibronectin receptor, and adhesion to fibronectin promotes cell survival in several conditions including hypoxia, serum starvation, and chemotherapy [43]. Furthermore, by using human breast cancer cell lines and a series of breast cancer biopsies from patients undergoing tamoxifen therapy, it was found that *α*6*β*4 integrin contributes to tamoxifen resistance *via* induction of ErbB-3 expression, which leads to an increase in AKT activation [44]. This seems to occur mainly in the estrogen-receptor-beta-1- (ERbeta1-) negative breast carcinomas. The authors found that, in these tumors, ErbB-3 inactivation inhibits AKT phosphorylation and induces apoptosis, thus favouring tamoxifen response. The analysis of human tumor biopsies revealed a significant correlation between *α*6*β*4 integrin/ErbB-3/phosphorylated-AKT signaling axis in ERbeta1-negative breast cancers derived from patients with lower disease-free survival. Together these studies indicate that ErbB oncogenic function in breast cancer cells is tightly regulated by integrin signaling, further supporting the role of integrins in breast cancer chemoresistance and survival. Furthermore, these studies show that in addition to DNA-damaging agents and microtubule-directed drugs, integrins in breast cancer cells also regulate resistance to drugs targeting specific oncogenes and steroid receptors.

 Fibronectin, collagens, and laminins are important matrix proteins of the breast cancer tumor microenvironment. The *α*2*β*1 integrin seems to be important for integrin-mediated attachment to collagen type I during metastasis of breast cancer cells to the bone [45, 46]. Of note, however, a recent study suggested that *α*2*β*1 integrin might suppress metastasis of breast cancer cells to the lymph nodes [47]. Gene expression analysis of drug-resistant MCF-7 breast cancer cells revealed that 25 genes encoding various ECM proteins (collagen, fibronectin, syndecan, laminin) and integrin subunits were upregulated in drug-resistant MCF-7 cells [48]. A clinical study also reported that increased *β*1 integrin expression is associated with decreased survival in invasive breast cancer [49], and inhibition of *β*1 integrin enhanced radiotherapy in human breast cancer xenografts [50].

**Figure 1:**
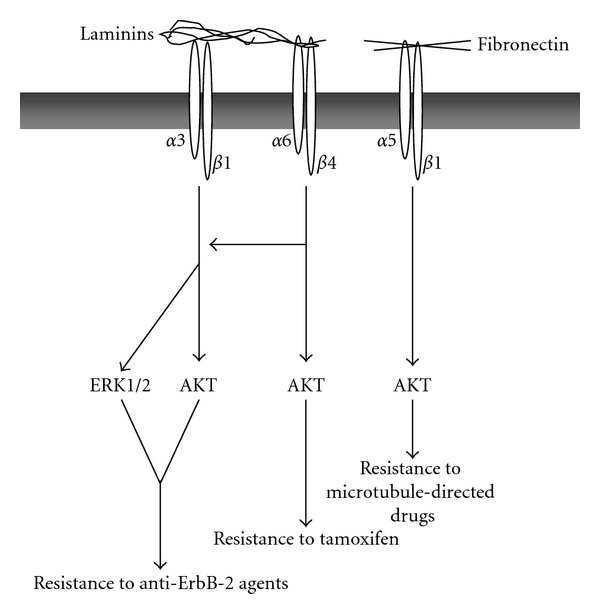
Laminin and fibronectin-mediated resistance to drug-induced apoptosis in breast cancer. Ligation of *α*3*β*1, *α*6*β*4, and *α*5*β*1 integrins with laminins and fibronectin, respectively, protects breast cancer cells from several cytotoxic agents *via* the activation of the PI 3-kinase/AKT and MAPK/ERK survival pathways.

 The studies described above indicate that the integrin-ECM signaling is a critical pathway in breast cancer resistance to chemotherapy. The role of a specific *β*1 integrin molecule is likely to be dependent on the differentiation status of the breast tumor (i.e., whether tumor is ErbB positive, invasive, etc.) and on the nature of the drug utilized. In this regard, laminin receptors are emerging as major integrins mediating breast cancer chemoresistance. A scheme summarizing the prosurvival role of laminin-binding integrins in breast cancer is depicted in Figure 1.

#### 4.1.2. Other Solid Tumors

Sethi et al. reported that adhesion of small cell lung cancer cells to fibronectin, collagen IV, and laminin inhibited apoptosis induced by chemotherapeutic agents including etoposide, cis-platinum, and daunorubicin as well as radiation [7]. The effect is mediated *via* *β*1 integrins among which *α*2*β*1, *α*3*β*1, *α*6*β*1, and *α*v*β*1 are the most expressed on these cells. Furthermore, the protective effect of *β*1 integrins is mediated through activation of the PI 3-kinase/AKT pathway, which inhibited drug-induced cell cycle arrest and caspase-3 activation [51]. The authors further showed that integrin-mediated activation of PI 3-kinase/AKT survival pathway overrides apoptosis by reducing the levels of p21 and p27 cell cycle inhibitors and by preventing downmodulation of cyclins E, A, and B. Consequently, chemotherapeutic agents are unable to induce G2/M cell cycle arrest, which is a necessary step in drug-induced apoptosis. In addition, the protective role of *β*1 integrin signaling did not occur at the level of DNA repair, indicating that the integrin-PI 3-kinase/AKT signaling pathway allows small cell lung cancer cells to survive chemotherapy despite DNA damage. Immunohistochemistry analysis of small cell lung cancer biopsies has revealed that these tumors produce large amounts of collagen IV, which could bind to *α*2*β*1 or *α*3*β*1 integrins, and fibronectin, which is a ligand for the *α*v*β*1 integrin [52, 53].

 A clinical study performed with transbronchial biopsies found that increased expression of *β*1 integrins correlates with chemoresistance and is a poor prognostic factor in small cell lung cancer [54]. Moreover, high levels of *β*1 expression and p53 were found to be a greater poor prognostic factor than clinical stage in small cell lung cancer [55]. Overexpression of *β*1 integrins has also been associated with the resistance of *non*-small-cell lung cancer to the tyrosine kinase inhibitor gefitinib, which targets the epidermal growth factor receptor tyrosine kinase [56]. Together these studies emphasize the critical role of *β*1 integrins in the malignancy and chemoresistance in lung cancer.

 Additional solid tumors also use attachment to ECM to escape apoptosis and chemotherapy. Thus, fibronectin inhibits ceramide- and docetaxel-induced apoptosis in the prostate cancer cell line DU145 *via* *β*1 integrins and insulin-like growth factor [57]. Parathyroid hormone-related protein in turn protects C4-2 and PC-3 prostate cancer cells from doxorubicin-induced apoptosis through integrin *α*6*β*4-mediated activation of the PI 3-kinase/AKT survival pathway [58]. Here, the parathyroid hormone-related protein/*α*6*β*4 integrin/PI 3-kinase/AKT signaling axis leads to an increase in the ratio of antiapoptotic to proapoptotic members of the Bcl-2 family and to activation of the transcription factor NF*κ*B, which is known to upregulate the expression of several antiapoptotic proteins.

 Interactions of pancreatic cancer cells with ECM including fibronectin, collagen type I, and collagen type IV decreased their sensitivity to cytotoxic drugs and promoted cell proliferation [59]. Furthermore, intrinsic chemoresistance to gemcitabine in these tumors correlates with constitutive laminin-induced FAK activation [60]. Activated FAK was shown to be required for activation of AKT, which mediates an increase in the expression of the anti-apoptotic protein survivin, and the inactivation of the Bcl-2 pro-apoptotic factor Bad *via* phosphorylation. A recent study also reported that pancreatic stellate cells can protect pancreatic cancer from radiotherapy-induced apoptosis through *β*1 integrin signaling involving FAK [61]. In addition, collagen I binding to *α*2*β*1 integrin has been shown to promote the malignant phenotype of pancreatic ductal adenocarcinoma and to protect from 5′-fluourouracil (antimetabolite drug)-induced apoptosis by upregulating the antiapoptotic protein Bcl-2 family member Mcl-1 [62]. Cell coculture models and human biopsies analysis have revealed that pancreatic tumors are enriched in the expression of several ECM proteins including collagens, fibronectin, and laminin [63]. The fibronectin receptor *α*5*β*1 also transduces the antiapoptotic effect of the adhesion molecule L1CAM (CD171) [64], and L1CAM/CD171 is associated with poor prognosis in several cancers such as colon and ovarian cancers [65]. Treatment of pancreatic cancer cells with chemotherapeutic drugs induces the expression of L1CAM, which binds to *α*5*β*1 integrin thus favoring chemoresistance. The prosurvival effect of L1CAM binding to the *α*5*β*1 integrin seems to be associated with activation of NF*κ*B and production of IL-1*β* [66].

**Figure 2:**
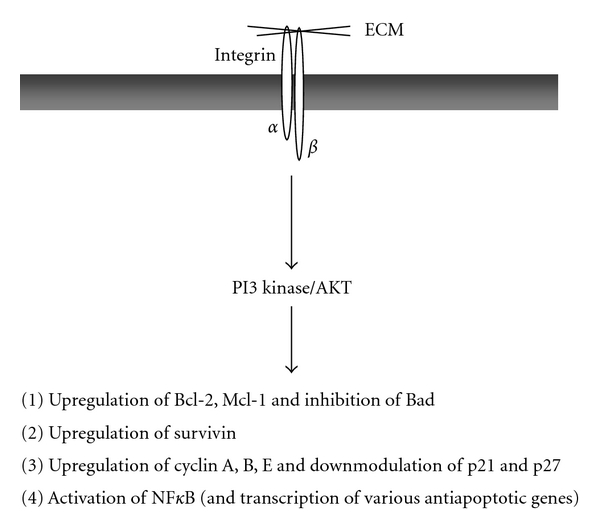
Role of PI 3-kinase/AKT in integrin-mediated drug resistance. Integrin/ECM interactions lead to the activation of PI 3-kinase/AKT which can regulate various downstream targets including proteins of the Bcl-2 family and of the IAP family, as well as cell cycle regulators. It also activates the transcription factor NF*κ*B, which is known to increase expression of several antiapoptotic proteins.

 The studies above point to a major role of PI3 kinase/AKT pathway in the chemoresistance of solid tumors. A scheme summarizing the mechanisms by which activation of PI3 Kinase/AKT promotes integrin-mediated chemoresistance is depicted in Figure 2.

### 4.2. Hematological Malignancies

Integrin-ECM signaling is also important for the survival of malignant cells of the hematopoietic origin. Several studies over the last decade have pointed to the *α*4*β*1 integrin as the principal ECM receptor involved in the survival and chemoresistance of multiple myeloma, myeloid, and B lymphoid malignancies. The role of the *α*4*β*1 integrin in these hematological malignancies has been outstandingly reviewed [11–14] and will not be the focus here. However, the *α*4*β*1 integrin does not seem to be as important for the survival of malignant T cells. The role of ECM in the survival and chemoresistance of T-cell neoplasms, which also grow in sites rich in ECM, remains poorly addressed. Several recent studies point to collagen-binding integrins as the major molecules involved in both normal and malignant T-cell survival. Below, we will discuss the expression and function of the *β*1 integrins in the T-cell lineage and how they contribute to the resistance of malignant T cells.

#### 4.2.1. Expression of ECM Receptors in the T-Cell Lineage

Normal T cells express several *β*1 integrins, which mediate their adhesion to fibronectin, laminin, and collagens [67]. Previously, *β*1 integrins and especially fibronectin receptors have been proposed to play an important role in T-cell adhesion and in T-cell costimulation and activation [68–70]. However, the collagen-binding integrins *α*1*β*1 and *α*2*β*1 have recently gained more attention as putative regulators of T-cell-mediated immunity and inflammation [67, 71, 72]. They are expressed only on effector T cells, which home to the inflamed tissues, whereas other *β*1 integrins such as fibronectin and laminin receptors are also found on naïve T cells. In addition, collagen is a more potent costimulatory molecule of human effector T cells than fibronectin [73], and we have shown that *α*2*β*1 integrin enhances the production of IFN*γ* and IL-17 in effector T cells [74, 75], two cytokines that play a crucial role in autoimmunity and tissue damage. T cells infiltrating inflamed sites in arthritis and other chronic inflammatory diseases can also express *α*1*β*1 and *α*2*β*1 integrins [76, 77]. Animal studies with mutant mice and blocking antibodies have demonstrated a critical role for the *α*2*β*1 integrin in the development of multiple sclerosis [78] and for both *α*1*β*1 and *α*2*β*1 integrins in delayed-type hypersensitivity and in arthritis [79, 80]. Together, these studies indicate that collagen-binding integrins can be crucial mediators of T-cell activation that is associated with the development of autoimmune diseases.

 Malignant T cells also express several *β*1 integrins. A number of T-cell acute lymphoblastic leukemia (T-ALL) cell lines such as Jurkat, HSB-2, and CEM express receptors for collagens, fibronectin, and laminins [81–84]. Previous reports have indicated that the collagen-binding integrin *α*1*β*1 is a predominant integrin expressed in cutaneous T-cell lymphomas and was proposed to be the major receptor mediating adhesion of these lymphomas to collagen I and collagen IV [85, 86]. Malignant cells such as T-ALL or T cell lymphoma also develop and grow in tissues rich in ECM such as the bone marrow, which is a privileged site for all hematological malignancies [12–14, 87, 88]. The niches for leukemia proliferation in the bone marrow are found in the epiphysial region [89], which consists of trabecular bone with spaces containing the red bone marrow. Immunohistochemical analysis has shown that collagen I is widely distributed in the trabeculae as well as throughout the marrow [90, 91], suggesting that collagen I, a major ECM component, could directly regulate interactions and anchorage of leukemia cells in their microenvironment. Clinical and experimental studies have shown that leukemia/lymphoma T cells can disseminate to organs such as liver, kidneys, and lungs [92–95], which are rich in ECM. Thus, ECM present in the microenvironment of lymphoid tumors is likely to regulate their survival.

#### 4.2.2. ECM/*β*1 Integrin in the Survival of Malignant T Cells

**(1) Regulation of Death Receptor-Mediated Apoptosis in T Cells**

The findings that anoikis can be mediated *via* activation of the death receptor pathway [96, 97] prompted us to examine if ECM can protect T cells from Fas-induced apoptosis, which is a major apoptotic pathway activated during immune response. Fas-induced apoptosis is critical in the maintenance of T-cell homeostasis at the end of immune response, and resistance to Fas-mediated death can contribute to inflammatory diseases and autoimmunity. Activation of the Fas pathway occurs in response to T cell-receptor- (TCR-) dependent stimulation also known as activation-induced cell death (AICD) [20, 98]. Restimulation of activated T cells through the TCR results in the transcriptional activation of *Fas* and its ligand (*Fas-L*) genes. Subsequently, ligation of Fas receptor with Fas-L induces apoptosis *via *DISC formation and caspase-8 activation.

Using the leukemic Jurkat T-cell line, which is sensitive to AICD and which constitutively expresses several *β*1 integrin members, we have demonstrated that engagement of the *α*2*β*1 integrin with collagen I inhibits AICD [15]. Ligation of *α*2*β*1 with collagen I or with an activating anti-*α*2 integrin monoclonal Ab (mAb) significantly reduced TCR-dependent apoptosis as well as PMA+Ionomycin-induced apoptosis, which is also partially mediated by the Fas-L/Fas death pathway. However, other matrix proteins such as fibronectin and laminin had no effect. Similarly, ligation of *α*1*β*1 with collagen IV also protected Jurkat T cells from AICD (our unpublished observations). The prosurvival effect of collagen-binding integrins observed in Jurkat T-cells also occurs in normal effector T cells [17, 99]. These observations indicate that collagen-binding integrins could promote autoimmune diseases by enhancing effector T cell survival and they could also promote T-cell malignancies.

Activation of Jurkat T cells with collagen I did not affect the expression of Fas receptor, which is expressed constitutively at high levels in these cells, but significantly reduced the transcriptional activation of the *Fas-L* gene upon TCR stimulation. Jurkat T cells activated with anti-TCR/CD3 mAb+collagen I are less efficient than Jurkat T cells activated by TCR/CD3 alone in killing the Fas-sensitive Hut-78 lymphoma used as target cells [15].

As noted above, focal adhesion kinase (FAK) has been shown to be central in integrin-mediated signaling and cell survival [100]. We have found that FAK is activated by both anti-CD3 mAb and collagen I, and expression of a dominant-negative form of FAK known as FRNK abrogated the protective effect of collagen I on Fas-L expression and AICD. These studies indicate that *α*2*β*1 integrin-mediated inhibition of AICD is dependent on the activation of FAK and inhibition of Fas-L. The potential role of integrin-mediated adhesion in the regulation of death ligands of the TNF family is also underscored in studies performed with adherent cells and could be one mechanism that regulates anoikis. We and others have demonstrated that the culture of endothelial and intestinal epithelial cells in suspension results in the transcriptional increase of death receptor ligands such as Fas-L and TRAIL, which subsequently triggers the activation of death receptor apoptotic cascades, thus contributing to the execution of anoikis [101–103].

We have subsequently investigated if activation of the *α*2*β*1 integrin with collagen I can directly regulate Fas signaling, thereby contributing to the inhibition of AICD. This was investigated in Jurkat T cells stimulated with the agonistic anti-Fas antibody CH11 to induce apoptosis directly through Fas receptor and independently from TCR stimulation [17]. In these conditions, we have found that collagen I significantly reduced Fas-induced apoptosis of Jurkat T cells. Interestingly, matrix proteins such as fibronectin and laminin that did not inhibit TCR-dependent apoptosis also had no effect on Fas-induced apoptosis [17]. Furthermore, inhibition studies with dominant negative forms and chemical inhibitors demonstrated that *α*2*β*1-mediated inhibition of Fas-induced apoptosis proceeds through activation of the MAPK/ERK survival pathway and inhibition of caspase-8 activation [17]. It is unclear whether the inhibition of caspase-8 was due to a reduction in DISC formation or to the reduction of proteolytic cleavage of procaspase-8. Along these lines, Eriksson's group demonstrated that activation of MAPK/ERK can block Fas-induced apoptosis by inhibiting the autoproteolytic activation of procaspase-8 [104], whereas Kaufmann's group showed that FADD, which is essential for DISC formation and caspase-8 activation, can be phosphorylated by MAPK/ERK, thereby contributing to the inhibition of DISC formation [105]. Collagen I may also modulate the localization of c-Flip, an endogenous inhibitor of caspase-8 activation at the DISC. This mechanism has been proposed to be downstream of the *α*4*β*1 integrin in the inhibition of Fas-induced apoptosis in monocytic U937 cells [19]. In addition, we found that collagen I also reduces TRAIL-induced apoptosis of Jurkat T cells [17]. Notably, the modulation of death receptor-mediated apoptosis by ECM also occurs in solid tumors. Fibronectin protects prostate cancer cells from TNF-induced apoptosis by activating AKT and upregulating the antiapoptotic protein survivin [16]. Ovarian cancer ascites inhibits TRAIL-induced apoptosis of ovarian cancer cells through *α*v*β*5 integrin-mediated FAK and AKT activation [18]. Together these studies indicate that, by protecting tumor cells from death receptor-mediated apoptosis, integrins can also contribute to tumor immune escape.

**Figure 3:**
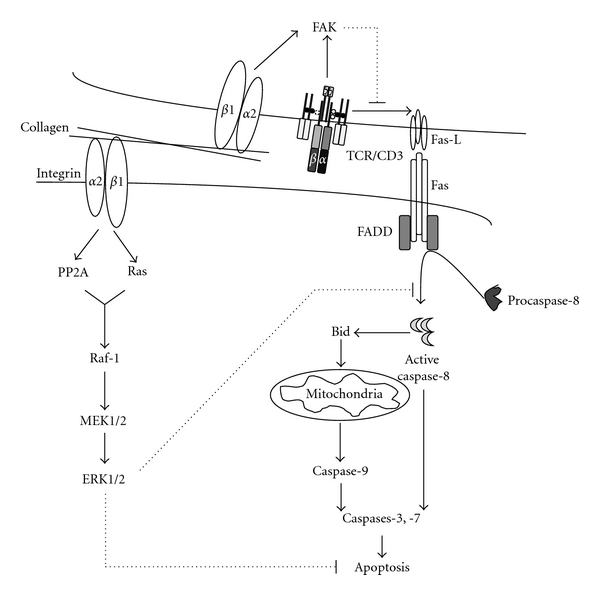
Model for collagen I/*α*2*β*1-dependent inhibition of AICD in T cells. Ligation of the *α*2*β*1 integrin in the context of TCR/CD3 activation leads to the synergistic activation of FAK, which leads to the reduction of TCR-induced Fas-L expression. Ligation of *α*2*β*1 with collagen I also induces the activation of the MAPK/ERK through the activation of Ras, protein phosphatase 2A (PP2A), and Raf-1. Active ERK inhibits Fas-induced signaling cascade by inhibiting caspase-8 processing and activation.

The findings that only collagen but not fibronectin or laminin receptors regulate death-receptor-induced apoptosis in malignant T cells is likely due to the differential ability of *β*1 integrin members to activate the MAPK/ERK survival pathway. Indeed, only collagen I was able to activate the MAPK/ERK pathway [17, 106]. We found that collagen I activates the MAPK/ERK pathway by activating Ras and protein phosphatase 2A (PP2A), which were both essential for collagen-mediated survival [17, 106]. Activation of PP2A is essential in the activation of c-Raf. The process by which PP2A activates Raf-1 seems to be exerted at the level of the Ser 259 inhibitory site [107, 108]. Dephosphorylation of this site by PP2A contributes to the release of Raf-1 from 14-3-3 inhibitory proteins and facilitates Raf-1's translocation to the membrane and interaction with active Ras, leading to Raf-1 activation. Interestingly, fibronectin also activates Ras in malignant T cells, but, unlike collagen I, it is unable to activate PP2A and c-Raf-1 [106]. Together these studies indicate that the differential ability of *β*1 integrin members to protect malignant T cells from Fas-induced apoptosis could at least partially be due to their ability to activate the PP2A/c-Raf/ERK pathway. Collagen-I-mediated activation of the MAPK/ERK pathway in Jurkat T cells also involves FAK [109]. A model summarizing how *α*2*β*1 integrin signaling inhibits Fas-induced apoptosis of the leukemic Jurkat T cells is depicted in Figure 3.

**(2) Role of ECM/Integrin Signaling in the Chemoresistance of Malignant T Cells**

In addition to Fas-induced apoptosis, evidence suggests that integrin signaling can also promote chemoresistance of malignant T cells. It has been reported that bone marrow stromal cells enhance the survival of T-ALL cell lines and blasts partially through the LFA-1 (*β*2 integrin)/ICAM-1 adhesion signaling system [110]. In addition, adhesion of Jurkat T cells to bone marrow stromal cells also provides them with a survival advantage against dexamethasone-induced apoptosis [111]. Given the functional role of *α*2*β*1 on death receptor-mediated apoptosis, we have studied if *α*2*β*1 integrin signaling modulates drug-induced apoptosis in malignant T cells. We showed that ligation of *α*2*β*1 integrin with collagen I significantly reduced doxorubicin-induced apoptosis of T-ALL cell lines Jurkat, HSB-2, and CEM [112]. We demonstrated that collagen I inhibited doxorubicin-induced apoptosis by inhibiting the expression of the receptor-activator of NF*κ*B-ligand (RANKL) [112]. RANKL is a cytokine of the TNF family, which binds to its receptor RANK as initially documented in osteoclast precursors and dendritic cells [113]. RANKL is expressed on osteoblasts and other mesenchymal cells as well as on activated T cells. During cognate cell-cell interactions, RANKL expressed on activated T cells induces the activation and survival of dendritic cells and osteoclast precursors [113]. Although RANK does not possess death domains [114] and thus is not coupled to DISC formation and caspase-8 activation, it has nevertheless been implicated in cell apoptosis [115]. Activation of RANK signaling in the absence of serum induces apoptosis of RAW macrophages [116]. Moreover, we and others have shown that RANKL is implicated in doxorubicin-induced apoptosis of leukemia T-cell lines [112, 117]. RANKL/RANK pathway participates in doxorubicin-induced apoptosis by contributing to the release of cytochrome c from the mitochondria [117]. Thus, by inhibiting the expression of RANKL, collagen I/*α*2*β*1 integrin signaling can contribute to reduced drug-induced cytochrome c release and protect the cells from chemotherapy.

**Figure 4:**
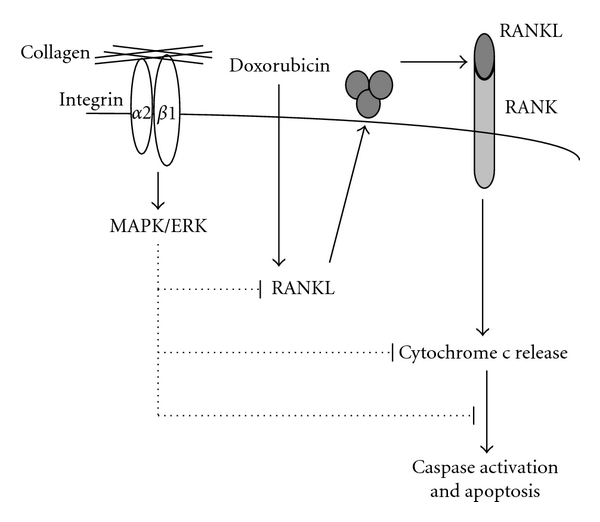
Model for collagen I/*α*2*β*1-mediated inhibition of drug-induced apoptosis in malignant T cells. Ligation of *α*2*β*1 integrin activates the MAPK/ERK, which blocks doxorubicin-induced RANKL expression. In turn, this leads to the reduction of cytochrome c release from the mitochondria and to the subsequent reduction of caspase activation and apoptosis.

Ligation of different *β*1 integrins with ECM proteins including collagen I and fibronectin was also shown to confer resistance to Ara-C- and radiation-induced apoptosis [118]. This was demonstrated upon overexpression of *β*1 integrins in HL-60 and Jurkat leukemia cell lines. The mechanism accounting for fibronectin/*β*1 integrin-dependent survival, at least in HL-60 cells, involves activation of the PI 3-kinase/AKT pathway, which inhibits caspase-8 activation. In our studies, we found that collagen I did not activate AKT in Jurkat T cells, and it was the collagen-mediated activation of MAPK/ERK that inhibited caspase-8 activation [17]. Further observations suggested that collagen-mediated MAPK/ERK also inhibited doxorubicin-induced RANKL expression and fibronectin, which is a weak activator of MAPK/ERK, did not protect from doxorubicin-induced apoptosis and had no effect on RANKL expression (our unpublished data). A model by which collagen inhibits doxorubicin-induced apoptosis of T-ALL cells is depicted in Figure 4.

The studies reviewed in this section indicate that collagen-binding integrins can protect malignant T cells from chemotherapy. Along these lines, Cleaver et al. recently reported that *α*2*β*1 integrin mRNA expression levels correlated with the resistance of pediatric T-ALL to the treatment with glucocorticoids [119]. Collagen I also protected Jurkat T cells from serum starvation-induced apoptosis by a mechanism involving activation of FAK/MAPK/ERK pathway [109]. Thus, it appears that collagen and its receptors *via* the activation of the MAPK/ERK pathway constitute a major survival pathway in malignant T cells. These findings are in contrast to those made in other hematological malignancies in which the *α*4*β*1 integrin is the main molecule mediating survival and drug resistance. This suggests that, depending on the cell type, hematological tumors could respond differently to their tissue microenvironment depending on the integrin expression profile and on the signaling events that become active in the cells upon integrin-ECM interaction.

## 5. Cooperation between Integrin and Growth Factor and Cytokine Receptors in Cell Survival and Chemoresistance

In addition to ECM, tumor cells also interact with soluble factors such as growth factors and cytokines that are present in their microenvironment. Integrins signal both independently and in collaboration with growth factor receptor signaling. The crosstalk signaling between integrins and growth factor and cytokine receptors has been investigated in a number of cell types and has been outstandingly reviewed [3, 120]. Integrins provide help to growth factor receptors by organizing signaling platforms for growth factor signaling and can directly activate growth factor receptors in a ligand-independent manner. In turn, activation of growth factor receptors can lead to increased integrin expression and avidity leading to enhanced cell adhesion and signaling. Integrins and growth factor receptors activate similar signaling pathways and likely cooperate regarding activation of the survival pathways MAPK/ERK and PI3 kinase/AKT [1, 3].

 Growing evidence indicates that the chemokine receptor CXCR4 cooperates with *β*1 integrins in mediating drug resistance of tumor cells. CXCR4 is the receptor for the stromal cell-derived factor-1 (SDF-1/CXC12), which is widely expressed in numerous tissues. It has been reported that small cell lung cancer cells express functional CXCR4 receptors and their activation with SDF-1 increases lung cancer cell adhesion to collagen I and fibronectin *via* *α*2*β*1, *α*4*β*1 and *α*5*β*1 integrins, respectively [121]. In addition, SDF-1 simulation enhances *β*1 integrin-mediated resistance against etoposide-induced apoptosis [121]. Mantle cell lymphomas also express high levels of chemokine receptors CXCR4, CXCR5, and integrin *α*4*β*1. These receptors were shown to be critical in adhesion of lymphoma cells to bone marrow stromal cells and also in their resistance against fludarabine-induced apoptosis [122]. Thus, CXCR4 inhibitors coupled with anti-*α*4*β*1 integrin antibodies were shown to abrogate both adhesion and chemoresistance of mantle cell lymphoma. Recently, it has been demonstrated that bone marrow stromal cell-induced chemoresistance of acute B-cell lymphoblastic leukemia (B-ALL) is mediated *via* a signaling complex composed of integrin *α*4*β*1, the chemokine receptor CXCR4, and the human ether-à-go-go-related gene channel (hERG1) [123]. Coculture of B-ALLs with bone marrow stromal cells induced the expression of the three receptors at the cell surface. The use of specific inhibitors indicated that all three receptors were necessary to protect B-ALLs from chemotherapy (doxorubicine, methotrexate, prednisone). This protective effect involves activation of both MAPK/ERK and PI 3-kinase/AKT survival pathways, which were shown to be activated by the assembled signaling complex. Interestingly, the use of hEGR-1 channel blockers was able to reverse drug resistance both in B-ALL blasts and in NOD/SCID mice engrafted with B-ALLs [123].

 Chemokine receptor (CXCR4) and integrin *α*4*β*1-mediated cooperative signaling seems also to involve activation of the Spleen tyrosine kinase (SYK) in chronic lymphocytic leukemia (CLL) [124]. In these studies, activation of SYK was reported to be essential for the inhibition of fludarabine-induced apoptosis, and the protective effect of SYK was found to be mediated through the phosphorylation of AKT and increased expression of the prosurvival Bcl-2 family member Mcl-1.

 Crosstalk between integrin receptors and the Wnt signaling pathway has also been shown to modulate the chemosensitivity of acute myeloid leukemia cells (AMLs) [125]. Adhesion of AMLs to fibronectin and Wnt antagonists induced independently AMLs' resistance towards daunorubicin. The protective effect of both pathways seems to require activation of the glycogen synthase kinase 3 beta (GSK3*β*) and NF*κ*B. These studies also established a link between adhesion and Wnt signaling in a coculture of the myeloid leukemic U937 cells and osteoblastic cells. Adhesion of U937 cells to osteoblastic cells was shown to induce the release of Wnt antagonist sFRP-1 from osteoblastic cells, which supported resistance to daunorubicin.

 Taken together, these studies indicate that several membrane receptors are likely to regulate integrin prosurvival function. Identification of these receptors will offer new possibilities for drug targeting and inhibition of integrin-mediated drug resistance.

## 6. Other ECM Receptors and Mechanisms in Tumor Cell Survival and Drug Resistance

Although not discussed above in great detail, several non-*β*1 integrins can also mediate drug resistance in tumor cells. For example, vitronectin through *α*v*β*3 and *α*v*β*5 integrins protects glioma cells from chemotherapy [9], and the angiogenic inducer CYR61, *via* *α*v*β*3, mediates resistance of breast cancer cell lines to taxol-induced apoptosis through activation of the MAPK/ERK pathway [126].

 Apart from the integrin-family of adhesion receptors, additional receptors expressed on mammalian cells also bind ECM. Herein, we will discuss the discoidin domain receptors (DDRs), which serve as receptors for several types of collagens. Originally, the DDR was described in breast cancer cells as an orphan tyrosine kinase receptor that has an extracellular discoidin-I-like domain similar to that found in the lectin of *Dictyostelium discoideum *[127]. DDRs are transmembrane tyrosine kinase receptors which are activated by various forms of collagens [128–130]. Two major related receptors, namely, DDR1 and DDR2, have been described, with DDR1 expressed as five isoforms (a–e) [131]. Ligation of DDRs with collagens leads to the dimerization of the receptor, which triggers the activation of the tyrosine kinase domain that leads to the autophosphorylation of tyrosine residues and to subsequent intracellular signaling [128–130]. The mechanism(s) of the crosstalk signaling between DDRs and integrins are unclear, but DDRs can bind and be activated by collagens independently from *β*1 integrins [132]. DDRs regulate several cellular functions including cell adhesion and migration, and proliferation.

 Growing evidence indicates that DDR1 is associated with tumorigenesis. DDR1 has been shown to be expressed in various human tumors including lung [133, 134], breast [127, 135], ovary [136, 137], and brain [138, 139] and to be associated with the production of metalloproteinases and cancer cell invasion of stroma tissues during metastasis [138, 140, 141]. DDR1 is one of the several tyrosine kinase genes that carries somatic mutations in small cell lung cancer and acute myeloid leukemia [142, 143]. In addition, DDR1 has been identified as a target for the Abl kinase inhibitor imatinib [134]. DDR1 also promotes cell survival in response to genotoxic stress. Irradiation treatment of p53-positive cancer cells induces in a p53-dependent manner the expression of DDR1, which activates the MAPK/ERK pathway leading to increased expression of p21, p19, and Bcl-xL and to cell survival [144]. In addition, DDR1 activation with collagen I also inhibits DNA-damage response in lung cancer cells *via* activation of Notch-1 signaling pathway [145]. Activation of DDR1 also protects breast cancer cells from DNA damage-induced apoptosis by inducing the expression of cyclooxygenase-2 through activation of NF*κ*B [146]. Although DDR1 signaling and physiological functions are still not well understood, these studies underscore the notion that these collagen receptors can also be important mediators of cancer cell invasion, survival, and chemoresistance.

 The use of three-dimensional (3D) cell culture models has revealed that, in addition to intracellular signaling activated by ECM receptors, resistance of tumor cells to chemotherapy can also be regulated by the physical barrier that the ECM presents to the tumor tissue, which could limit the penetration of the drugs into tumor cells. Several studies have reported that cell-adhesion-mediated drug resistance in various tumor cell spheroids models is more profound than that seen in tumor cells grown on 2D matrices [147–152]. The 3D form of collagen I has been appreciated as a major barrier contributing to chemoresistance [153–157]. However, care should be taken when interpreting these results, as 3D architecture, compared to 2D culture conditions, may also contribute to differences in intracellular signaling that can in turn affect tumor cell survival and drug responsiveness.

 The tumor tissue *in vivo* is characterized by a high interstitial fluid pressure, which is due in part to tumor-stromal production and organization of collagen I. This high interstitial fluid pressure is a major factor in the formation of tumor barrier to transcapillary transport [158], and has been shown to exist in several types of tumors such as breast and colorectal cancers [159, 160], metastatic melanoma [161, 162], and head and neck carcinoma [163]. It is inversely correlated with intratumoral uptake of various molecules such as antibodies [164] and chemotherapy [165]. In this regard, the intratumoral collagen I of human ovarian cancer xenografts (SKOV-3 and OVCAR-3) reduces the transport of intraperitoneally injected antibody into the tumor parenchyma, and treatment with collagenases has been shown to enhance antibody penetration in the tumors [166]. Targeting tumor-associated fibroblasts also improves cancer chemotherapy by increasing intratumoral drug uptake [167]. These studies suggest that the interstitial matrix barrier may need to be overcome before effective drug or antibody delivery can take place and that such barrier contributes to the complex role that cell adhesion and ECM play in tumor chemoresistance.

## 7. Integrins in Cancer Stem Cells

Cancer stem cells also defined as tumor-initiating cells are a minor subpopulation of tumor cells that are critical for tumor maintenance, metastasis, and therapeutic resistance. Recent studies have shown that adhesion to ECM can also regulate the tumorigenesis of these cancer subpopulations by regulating their homing to their niches, their maintenance in the niche and by regulating their proliferation and self-renewal [168]. One important molecule that has been described in this process is the CD44; the receptor for hyaluronic acid. CD44 is expressed on several types of cancer stem cells including breast, prostate, glioma as well as on leukemia initiating cells [169]. CD44 can regulate cancer stem cell tumorigenesis by promoting matrix assembly, allowing the local concentration of glycosaminoglycan-associating proteins such as FGF2 and VEGF and promoting migration and the epithelial-mesenchymal transition, which is a critical step in invasion and metastasis [169]. The *α*6 integrin also regulates self-renewal and proliferation of glioblastoma stem cells [170], and CD44 and *α*2*β*1 integrin regulate tumorigenesis of prostate cancer stem cells [171, 172]. Expression of integrin *α*v is also required for the acquisition of a metastatic stem/progenitor cell phenotype in human prostate cancer [173], and vitronectin/*α*v*β*3 interaction also induces breast and prostate cancer stem cell differentiation and tumor formation [174].

Although the mechanisms accounting for the resistance of cancer stem cells are not yet clear, the rapid drug elimination by drug transporters could explain their resistance to chemotherapy [175, 176]. CD44 can also contribute to this process. Indeed, CD44 has been reported to upregulate the expression of the Pg-p drug transporter by a positive feedback involving hyaluronan, PI3 Kinase, and ErbB2 [177]. CD44 can also regulate drug resistance by modulating glucose metabolism in cancer cells [178]. In addition, it has been reported that the Y-box binding protein-1 (YB-1), an oncogenic transcription/translation factor, which is expressed in more than 40% of breast cancers, induces the expression of CD44 and of *α*6 integrin, which led to enhanced self-renewal, mammosphere growth, and resistance to paclitaxel treatment [179]. The *β*3 integrin has been involved in the survival of breast tumor-initiating cells [180]. These studies suggest that adhesion of cancer stem cells to ECM is likely to contribute to their drug resistance as well. However, additional studies are required to understand the role and the underlying mechanisms of integrins in drug resistance of cancer stem cells.

## 8. Concluding Remarks

It is recognized that the tumor microenvironment plays a critical role in cancer cell survival and progression. The studies reviewed here support a general function of ECM/integrin signaling in tumor cell survival and in the development of chemoresistance. ECM/integrin signaling pathway can, therefore, constitute a major pathway contributing to minimal residual disease and patient relapse, and its targeting could significantly improve anticancer therapy and patient survival.

 Because of their known role in angiogenesis and migration, several integrin inhibitors are being developed as therapeutic agents for cancer [181]. A humanized anti-*α*v*β*3 antibody and cyclic peptide inhibitors of integrin *α*v*β*3/*α*v*β*5 as well as a humanized anti-*α*5*β*1 antibody are currently in clinical trials in several types of cancer such as glioblastoma, breast cancer, and melanoma. An additional approach to alleviate cell-adhesion-mediated drug resistance is the development of new drugs of which the cytotoxic effects are not modulated by ECM/integrin signaling. In this regard, the proteosome inhibitor bortezomib, the *β*1 integrin antagonist HYD1, and statins can represent promising drugs. These three agents overcome cell-adhesion-mediated drug resistance in multiple myeloma either through the downmodulation of *α*4*β*1 integrin expression, or in the case of statins, *via* geranylgeranylation of Rho protein and activation of Rho kinase [182–184]. Future studies should also investigate if anti-integrin antagonists used in combination with current chemotherapeutic drugs can be beneficial in preventing drug resistance and patient relapse. Further understanding of tumor-stroma interactions, the contribution of integrins to cancer stem cell survival and drug resistance as well as the determination of the complete integrin “signalosome” may lead to the identification of novel therapeutic targets.
